# Supplementary material for: Of Money and Men: A Scoping Review to Map Gender Barriers to Immunization Coverage in Low- and Middle-Income Countries
Source: Vaccines (Basel). 2024 Jun 5;12(6):625. doi: 10.3390/vaccines12060625 (PMC11209140; doi:10.3390/vaccines12060625)
Supplement: Supplementary file 1 [file vaccines-12-00625-s001.zip › vaccines-3012730-supplementary.pdf]

Gender Influencers on Immunization	Sub-theme	Key points	Geographies/ References
**Intent -** the demand for vaccines that would result in vaccination in the absence of other barriers.			
1. Women's autonomous decision making	Lack of decision-making over health	- In many settings women rely on their husband's or an elder's permission to seek healthcare services including immunization.<br>- Women with high household decision-making are more likely to have fully immunized children.<br>- Women who make decision jointly with their husbands are more likely to have fully immunized children than when husbands make decision alone.<br>- Some women who oppose their husband's decision face increased risk of intimate partner violence.	Africa (n=37)<br>South Asia (n=16)<br>Cross-country (n=3)<br>(8,12,17–59)
2. Past experiences with the health system	Negative Experiences	-Some women reported being shamed / bullied by health workers if they missed a prior appointment, forgot the child's vaccine card, or if they or their child appeared dirty and/or malnourished.<br>- Caregivers were not always provided with complete information about the vaccination, including likely side effects and how best to alleviate those side effects.<br>- Caregivers who experience disrespectful treatment are least likely to return to the health system.	Burkina Faso, DRC, Ethiopia Gabon, Nigeria, Mozambique, Pakistan, Timor-Leste, Uganda (n=8)<br>(25,38,55,61,71,72,76,84)
	Engagement with ANC	Women who attend ANC are more likely to have fully immunized children than women who do not.	Afghanistan, Ethiopia, Nepal, Senegal (n=8)<br>(16,27,50,68,80,81,87,91)
3. Gendered myths, misconceptions	Fears of infertility	-Caregivers and health workers expressed concerns that vaccines (including HPV, COVID-19, H1N1, and childhood immunizations) could cause infertility.	Burkina Faso, Kenya, Malawi, Morocco, Tanzania, Zambia (n=7)(18–20,24,30,61,62)

	Promotion of earlier / increased sexual activity	-Caregivers feared that HPV immunization would result in earlier sexual debut / increased sexual activity for adolescent girls.	Ethiopia, Ghana, India, Malawi, Papua New Guinea, South Africa, Zimbabwe (18,35,54,58,63–65)
**Access -** ability or inability to successful carry out the transaction of vaccine utilization.			
1. Time poverty	Competing and gendered demands on time	-Women face competing demands on their time including employment and gendered expectations of caregiving and household labor. This reduces their time for immunizations.<br>- Men's limited contribution in unpaid domestic work exacerbates the demand on women's time.	Burkina Faso, Ethiopia, Nigeria, DRC, Mozambique, Sierra Leone, South Africa, India, Philippines, Somalia, Malaysia, Philippines, Pakistan, Timor-Leste, Uganda, Malawi, Gabon (18,23,25,40,41,55,61,66,71–74,101) (21,35,43,44,54,72,73)
	Distance to facilities	- Timing of (schedule) and distance to services can exacerbate this challenge. This effect is worse for low-income women who are socially isolated.	Gabon, Malawi, Nigeria, Malaysia, South Africa, Uganda, Guinea, Malawi, Ethiopia, DRC, Mozambique, Bangladesh (17,25,28,36,66,71,77–82).
2. Direct costs	Costs of vaccines, transportation to services, and illicit fees.	-Women reported that the cost of vaccine, transportation costs to access facilities, and illicit fees for services were barriers to immunization.	DRC, Ethiopia, Gabon, Guinea, India, Kenya, Malaysia, Mozambique, Nigeria, Pakistan, Philippines, South Africa, Uganda, Zambia, Zimbabwe (19,23,25,28,29,36,58,62,71,74,79,84–87)

	Financial Agency	-Women lack financial agency, relying on their husbands to provide the funds and/or approve use of funds for immunization.<br>- Women with their own income and discretion about spending it had increased odds of their children being fully immunized.	DRC, Ethiopia, Gabon, Mozambique, India, Nigeria, Uganda (15,16,25,34,38,66,71,78,81,88)
**Readiness -** encompasses the health system's supply of vaccine services to adequately meet demand.			
1. Vaccinators / Health care providers	Lack of women vaccinators/preference for women vaccinators	-A lack of women vaccinators leads to increased coverage inequities, and many men prefer women/daughters are vaccinated by women.	Bangladesh, DRC, Ethiopia, India, Nigeria, Pakistan, Somalia (10,33,43,57,89)
	Women workers' occupational concerns	- Many women health workers experience safety issues, harassment, and low or late remuneration for their services.	Afghanistan, Bangladesh, DRC, Ethiopia, India, Nigeria, (35,90)
2. Health care facilities	Gender unintentional facilities	-Lack of privacy and gender-responsive facilities (i.e. functional and separate washrooms and security for transgender individuals) is a barrier.	Bangladesh, Pakistan (33,84)
	Excessive wait times	- Excessive wait times result in children not receiving immunizations and/or caregivers not being willing to return.	Burkina Faso, DRC, Guinea, Ethiopia, Mozambique, Nigeria, Uganda (25,36,61,66,76,80).
3. Vaccine availability	Vaccine stockouts	-Unavailability of vaccines can lead to pessimism and future nonadherence.<br><br>-Restrictive vial opening policies result in delayed vaccination and increased frustration among caregivers.	Burkina Faso, Ethiopia, Gabon, Guinea, Nigeria, Papua New Guinea, Tanzania, Uganda (20,36,61,64,66,71,72,76).
